# Supplementary material for: Efficacy and safety of Galgeun-tang-ga-cheongung-sinyi for nasal congestion with common cold: A study protocol for randomized, double-blind, placebo-controlled, parallel, multicenter clinical trial
Source: PLoS One. 2026 Feb 5;21(2):e0342433. doi: 10.1371/journal.pone.0342433 (PMC12875452; doi:10.1371/journal.pone.0342433)
Supplement: S2 File — (PDF) [file pone.0342433.s002.pdf]

Clinical Trial Protocol

Efficacy and safety of Galgeun-tang-ga-cheongung-sinyi for nasal congestion with common cold: a randomized, double-blind, placebo-controlled, parallel, multicenter clinical trial

Protocol No.	KIOM_2024_CC_GGTCS
Phase of Study	Investigator-initiated clinical trial
Version	1.3
Date	2025.01.20
Principle Investigator	1. Yang-Chun Park/ Daejeon University Daejeon Korean Medicine Hospital<br>2.Beom-Joon Lee/ Kyung Hee University Korean Medicine Hospital<br>3.Jun-Yong Choi/ Pusan National University Korean Medicine Hospital

The clinical study will be conducted in accordance with the protocol, guideline (ICH E6) regarding Good Clinical Practice (GCP) of International Council for Harmonisation of Technical Requirements for Pharmaceuticals for Human Use (ICH), and related law

Table of Contents

Protocol version history 5
List of Abbreviation 6
SYNOPSIS 7
Schedule Summary 14
1. Title and Phase of the Clinical Trial 16
 1.1. Title of the Clinical Trial 16
 1.2. Phase of the Clinical Trial 16
2. Clinical Trials Institutions, Central Laboratory, Principal Investigators 16
 2.1. Names and Addresses of Clinical Trials Institutions 16
 2.2. Principal Investigators 16
 2.3. Monitoring 16
3. Background 17
4. Objectives 18
5. Participants 19
 5.1. Inclusion Criteria 19
 5.2. Exclusion Criteria 19
 5.3. Sample Size 20
 5.4. Recruitment 21
6. Trial Design 22
 6.1. Period of Trial 22
 6.2. Procedure 22
 6.3. Randomization 23
 6.4. Blinding 23
7. Dropout and Early Termination Criteria 24
 7.1. Dropout 24
 7.2. Early Termination 25
 7.3. Completion Criteria 26
8. Investigational Product 26
 8.1. GGTCS 26
 8.2. Placebo 27
 8.3. Management of Investigational Product 27
 8.4. Return and Disposal 29
9. Methods and Administration 30
 9.1. Investigational Product Administration 30
 9.2. Concomitant and Prohibited Medications 30
 9.3. Rescue Medication 30
 9.4. Compliance Assessment 31
10. Procedure 31

10.1. Timetable 31
10.2. Observation and Evaluation by Visit Schedule 33
10.2.1. Visit 1 (Screening Visit/Day -1 ~ Day 0) 33
10.2.2. Visit 2 (Baseline/Day 0) 35
10.2.3. Visit 3(Final visit/Day 7-10) 36
10.2.4. Additional Visits 37
10.2.5. Monitoring for Symptom Worsening 37
10.3. Outcome Measures 38
10.3.1. Primary Outcome Measures 38
10.3.2. Secondary Outcome Measures 39
10.4. Safety Assessment 41
10.5. Exploratory outcome measures 41
10.5.1. Questionnaire for Common Cold Pattern Identification 41
10.6. Adverse Events and Serious Adverse Events 42
10.6.1. Definition of Adverse Events 42
10.6.2. Definition of Serious Adverse Events 42
10.6.3. Classification of Adverse Events 43
10.6.4. Follow-up of Adverse Events 45
10.6.5. Reporting of Adverse Events 45
11. Data Analysis and Statistical Analysis 47
11.1. General Principles of Statistical Analysis 47
11.1.1. Definition of Analysis Sets 47
11.1.2. General Principles of Analysis 47
11.1.3. Handling of Missing Data 48
11.1.4. Analysis of Demographic and Baseline Data 48
11.2. Statistical Analysis of Outcome Measures 48
11.2.1. Primary Outcome Measures 48
11.2.2. Secondary Outcome Measures 48
11.2.3. Exploratory Outcome Measures 49
11.3. Statistical Analysis of Safety Assessment 49
11.4. Collection and Management of Clinical Trial Data 49
11.5. Storage and Confidentiality 49
11.5.1. Storage of Clinical Trial Documents and Data 49
11.6. Confidentiality of Clinical Trial Data and Subject Records 50
12. Ethical Considerations and Administrative Procedures 50
12.1. Subject Consent Procedures 50
12.2. Measures for the Ethical Conduct of the Trial and Protection of Subject Safety 50
12.2.1. Clinical Trial Site 51
12.2.2. Approval and Amendment of the Clinical Trial Protocol 51
12.2.3. Familiarization with the Clinical Trial Protocol 51

12.3. Participant Compensation Policy 51
12.4. Confidentiality of Subject Records 51
12.5. Protective Measures for Vulnerable Subjects 52
12.6. Monitoring and Quality Control 52
12.7. Reporting of Trial Results 53
12.8. Publication of Trial Results 53
13. Other Necessary Matters for the Safe and Scientific Conduct of the Clinical Trial 53
13.1. Compliance with Laws, Audits, and Prohibitions 53
13.2. Research Data Sharing Plan 54
14. References 54

Protocol version history

No	Version No.	Version Date	Updates
1	1.0	2024.09.23	Original establishment
2	1.1	2024.12.16	Change in study title and objective, addition of a secondary efficacy endpoint (nasal congestion symptom score)
3	1.2	2025.01.06	Addition of a secondary efficacy endpoint (common cold recovery status)
4	1.3	2025.01.20	Change in discontinuation criteria, addition of rescue medication dosage

List of Abbreviation

Abbreviation	Definition
AE	Adverse Event
ALT	Alanine Aminotransferase
ANCOVA	Analysis of Covariance
AST	Aspartate Aminotransferase
BUN	Blood Urea Nitrogen
CRF	Case Report Form
EQ-5D-5L	5-level EuroQol 5-dimensional questionnaire
FAS	Full Analysis Set
HCG	Human Chorionic Gonadotropin
ITT	Intention-to-treat
PNS	Paranasal sinuses
PP	Per Protocol
RBC	Red Blood Cell
VAS	Visual Analogue Scale
WBC	White Blood Cell
WURSS-21-K	Wisconsin Upper Respiratory Symptom Survey, Korean version

SYNOPSIS

Title	Efficacy and safety of Galgeun-tang-ga-cheongung-sinyi for nasal congestion with common cold: a randomized, double-blind, placebo-controlled, parallel, multicenter clinical trial
Objective	To evaluate and compare the efficacy and safety of GGTCS compared with placebo for nasal congestion with common cold.
Design	Phase: Investigator-initiated trial<br>Design: Randomized, double-blind, placebo-controlled, parallel, multicenter clinical trial
Investigational Site	Daejeon University Daejeon Korean Medicine Hospital/75, Daedeok-daero 176beon-gil, Seo-gu, Daejeon, South Korea<br>Kyung Hee University Korean Medicine Hospital/23, Kyungheedae-ro, Dongdaemun-gu, Seoul, South Korea<br>Pusan National University Korean Medicine Hospital/20, Geumo-ro, Mulgeum-eup, Yangsan-si, Gyeongsangnam-do, South Korea
Principal Investigator	Yang-Chun Park/Daejeon University Daejeon Korean Medicine Hospital<br>Beom-Joon Lee/Kyung Hee University Korean Medicine Hospital<br>Jun-Yong Choi/Pusan National University Korean Medicine Hospital
Target Disease	Patients with nasal congestion due to common cold
Duration	During 24 months from IRB approval date
Investigational Product	1. GGTCS<br><table><tr><td>Name</td><td>Kyungbang Galgeun-tang-ga-cheongung-sinyi</td></tr><tr><td>Manufacturer</td><td>Kyungbang Pharm Co.</td></tr><tr><td>Ingredients and Content</td><td>Per single dose (3.0g): Puerariae Radix 2.67g, Ephedrae Herba 1.33g, Cinnamomi Ramulus 1.00g, Paeoniae Radix 1.00g, Glycyrrhizae Radix et Rhizoma 0.67g, Zingiberis Rhizoma Crudus 0.33g, Zizyphi Fructus 1.33g, Cnidii Rhizoma 1.00g, Magnoliae Flos 1.00g</td></tr><tr><td>Formulation and Appearance</td><td>Light brown granules</td></tr></table><br>2. Placebo: Granules identical in appearance to the GGTCS.
Dose of Investigational Product	1. GGTCS Group: Orally administer 1 sachet of the investigational product (1 sachet of GGTCS) three times a day, before or between meals.<br>2. Placebo Group: Orally administer 1 sachet of the control product (1 sachet of placebo) three times a day, before or between meals.

<table>
<tr><td>Duration of Administration</td><td>Maximum 7 days</td></tr>
<tr><td>Methods</td><td>After voluntarily signing the informed consent form, subjects will be assessed for eligibility based on the inclusion and exclusion criteria. Only eligible subjects will be randomized to either the test group or the control group in the order of their enrollment. The assigned subjects will take the investigational product (test drug or placebo) for a maximum of 7 days.
The study will investigate the following assessments of the the WURSS-21-K total score, symptom score, quality of life score, Nasal Congestion Severity Score, Nasal Symptom Score VAS, Systemic Symptom Score VAS, and duration of cold symptoms in each time points, Global Evaluation of Efficacy score on Day 7, EQ-5D-5L score on Day 7 from baseline, and the change in WURSS-21-K total score according to the Cold Pattern Identification (Wind-Cold, Wind-Heat, or other types).

■ Symptom improvement effect
■ Quality of life evaluation
■ Comparison of effects based on Korean medicine diagnostic criteria

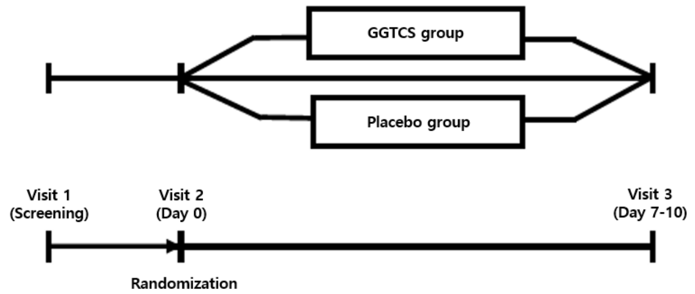
</td></tr>
<tr><td>Sample Size</td><td>
<table>
<tr><td></td><td>GGTCS group</td><td>Control group</td><td>Total</td></tr>
<tr><td>Final efficacy population (PP)</td><td>70</td><td>70</td><td>140</td></tr>
<tr><td>Population considering dropout rate (20%)</td><td>88</td><td>88</td><td>176</td></tr>
</table>

- Target number of subjects per institution
<table>
<tr><td>Daejeon University Daejeon Korean Medicine Hospital</td><td>44</td><td>44</td><td>88</td></tr>
<tr><td>Kyung Hee University Korean Medicine Hospital</td><td>22</td><td>22</td><td>44</td></tr>
<tr><td>Pusan National University</td><td>22</td><td>22</td><td>44</td></tr>
</table>
</td></tr>
</table>

	Korean Medicine Hospital | | |
Inclusion criteria	1. Men and women aged between 19 and 75 years<br>2. Diagnosed with the common cold, symptoms occurring within 72 hours of the start of the trial<br>3. More than two symptoms related to the common cold including runny nose, nasal congestion, sneezing, sore throat, cough, headache, chills, and body aches<br>4. Nasal congestion severity score greater than 2<br>5. Those who voluntarily decide to participate in this clinical trial and give written informed consent to abide by the precautions after listening a detailed explanation of this clinical trial and fully understanding it.
Exclusion criteria	1. Those with sinusitis, allergic rhinitis, pneumonia, influenza, COVID-19, cough or sore throat with sudden fever above 38 ℃, bronchitis, otitis media, tonsillitis (if accurate examination is necessary, paranasal sinuses (PNS) view or chest X-ray will be conducted)<br>2. Diagnosis of chronic respiratory disease (chronic obstructive pulmonary disease, bronchial asthma, bronchiectasis, interstitial lung disease, and other chronic respiratory diseases)<br>3. Those who had taken antibiotics, antivirals, steroids, nasal decongestants, antihistamines, antitussives, expectorants, or other medications to alleviate symptoms of the common cold within 1 week of screening<br>4. Liver or renal impairment (ALT, AST, and creatinine ≥ 3 times the upper normal limit at screening)<br>5. Those with generic disorders such as galactose intolerance, Lapp lactase deficiency, or glucose-galactose malabsorption<br>6. Pseudo-aldosteronism or myopathy induced by hypokalemia<br>7. Comorbidities that interrupt the treatment of cancers or clinically significant disorders of the kidney, liver, psychiatric system, cardiovascular system, respiratory system, endocrine system, or central nervous system thus interfering with the assessment of efficacy and safety of the investigational drug or completion of the clinical study<br>8. Unregulated hypertension (systolic blood pressure ≥ 160 mmHg or diastolic blood pressure ≥100 mmHg)<br>9. Unregulated diabetes mellitus (fasting blood sugar ≥ 180 mg/dL)<br>10. History of hypersensitivity reaction to materials of drugs used in this clinical trial<br>11. History of alcoholism or substance abuse

	12. Current smokers or those with history of smoking more than 30 packs/year<br>13. Pregnant or lactating women<br>14. Those who do not use medically acceptable contraception (e.g., intrauterine device with proven pregnancy failure rate in spouse or partner, simultaneous use of barrier method for men or women with spermicide, or surgical procedures, such as vasectomy, tubectomy, tubal ligation, or hysterectomy for oneself or one's partner) during the clinical trial<br>15. Those who participated in other clinical trials within 30 days before participation in this clinical trial<br>16. Those determined by investigators to be ineligible to participate in this trial
Prohibited Concomitant Medications	1. Antibiotics, antiviral agents, steroids, decongestants, antihistamines, antitussives/expectorants, and other medications expected to relieve nasal congestion<br>2. Medications administered for the same therapeutic purpose as the investigational product or those that may affect the improvement of nasal congestion symptoms.<br>3. Medications containing the same active ingredients (Puerariae Radix, Ephedrae Herba, Zizyphi Fructus, Cinnamomi Ramulus, Paeoniae Radix, Glycyrrhizae Radix et Rhizoma, Zingiberis Rhizoma Crudus, Cnidii Rhizoma, and Magnoliae Flos), even if they are not used for the same therapeutic purpose as the investigational product.
Efficacy Outcome Measures	• **Primary Outcome Measures**<br>1. Change in the WURSS-21-K total score (symptom score + quality of life score) from Baseline to Day 7<br>• **Secondary Outcome Measures**<br>1. Change in the WURSS-21-K total score (symptom score + quality of life score) from Baseline to each time point (excluding Day 7)<br>2. Change in the WURSS-21-K symptom score from Baseline to each time point<br>3. Change in the WURSS-21-K quality of life score from Baseline to each time point<br>4. Change in the Nasal Congestion Severity Score from Baseline to each time point<br>5. Change in the Nasal Symptom Score VAS from Baseline to each time point<br>6. Change in the Systemic Symptom Score VAS from Baseline to each time point

	7. Duration of common cold symptoms<br>8. Common cold recovery status on Day 7<br>9. Global Evaluation of Efficacy score on Day 7<br>10. Change in the EQ-5D-5L score from Baseline to Day 7<br>• **Exploratory Outcome Measures**<br>1. Change in the WURSS-21-K total score according to the common cold pattern identification (Wind-Cold type, Wind-Heat type, or Other types).
Safety Outcome Measures	1. Adverse events<br>2. Vital signs<br>3. Laboratory tests
Statistical Analysis	• **General Principles of Statistical Analysis**<br>1. Definition of Analysis Sets<br>For the analysis of data from this clinical trial, efficacy analysis will primarily use the Full Analysis Set (FAS) as the main method, and the Per Protocol Set (PPS) analysis will be used as a supplementary confirmation when necessary.<br><br>1) The FAS is defined according to ITT principle, which includes all randomized subjects who received at least one dose of the investigational product and have at least one post-baseline efficacy measurement. All data obtained from these subjects will be included in the analysis. However, subjects who do not meet a pre-established criteria will be excluded. The exclusion criteria for the FAS are as follows:<br>i. Subjects who violate the inclusion or exclusion criteria<br>ii. Subjects who did not take the investigational product even once<br>iii. Subjects who did not provide any data after screening<br>2) The PPS analysis set consists of a subset of subjects from the FAS who have a drug compliance rate of at least 70% of the total investigational product dose, completed the entire trial process as specified in the protocol, and have no major protocol violations that could affect the results. The definition of major violations is based on the content described in section 7.1 of this protocol.<br><br>For the safety analysis of the data obtained from this clinical trial, all data from subjects who received at least one dose of the investigational product after randomization and underwent a safety evaluation will be included in the analysis. For safety analysis, subjects will be analyzed in the group they actually received treatment in.

2. General Principles of Analysis
   1) All statistical analyses of the clinical trial results will be based on two-sided testing, with a significance level of 5%.
   2) The statistical software used for analysis will be SAS® Version 9.4 (SAS Institute Inc., Cary, NC).
   3) For continuous data, the mean and 95% Confidence Interval will be presented. For categorical data, frequency and percentage (%) will be presented.
   4) If necessary, a subgroup analysis will be performed by categorizing subjects based on their initial characteristics from the screening or baseline visits.

3. Handling of Missing Data
   In this study, missing values will be imputed using the Last Observation Carried Forward (LOCF) method. This imputation will only be applied to efficacy endpoints in the FAS.

4. Analysis of Demographic and Baseline Data
   Descriptive statistics for demographic characteristics and pre-treatment subject characteristics will be presented for each group. For continuous variables, the mean and confidence interval will be presented, and an independent t-test will be performed. For categorical variables, frequency and proportion will be presented, and a Chi-square test or Fisher's exact test will be performed.

• **Statistical Analysis of Outcome Measures**

1. Primary Outcome Measures: Change in WURSS-21-K Total Score (Symptom Score + Quality of Life Score) from Baseline to Day 7
   - Research Hypothesis: The change in the WURSS-21-K total score from baseline to Day 7 in the test group is not equal to the change in the WURSS-21-K total score from baseline to Day 7 in the control group.

   - The primary outcome measures will be analyzed using an ANCOVA model, with the groups as a fixed effect and the baseline WURSS-21-K total score as a covariate. If necessary, variables that show a statistical difference in demographic characteristics or variables that may affect cold symptoms can be included as covariates. The least-square mean (LSM) for the difference between the test group and the control group will be calculated, and the 95% confidence interval and p-value for this difference will be presented.

2. Secondary Outcome Measures
   1) The changes in the following secondary outcome measures from baseline to Day 7 will be tested using the same method as the primary efficacy endpoint: Nasal Congestion Severity Score, Nasal Symptom Score VAS, Systemic Symptom Score VAS, WURSS-21-K symptom score, WURSS-21-K quality of life score, and EQ-5D-5L score. Additionally, the pre- and post-treatment differences within each group will be analyzed using a Student's paired t-test, depending on the normality of the data.
   2) To compare the differences in trends between the groups, Repeated Measures Analysis of Variance (RM ANOVA) will be used, with Dunnett's procedure for multiple comparisons (comparing each time point against baseline).
   3) The Global Evaluation of Efficacy satisfaction and cold recovery status at Day 7 will be analyzed using the Chi-square test. However, if the Global Evaluation of Efficacy satisfaction score is treated as a continuous variable, an Independent t-test or Wilcoxon rank-sum test will be performed.

3. Exploratory Outcome Measures
   - The analysis methods for comparing different syndromes will be the same as those for the efficacy outcome measures.

• **Statistical Analysis of Safety Assessment**

Safety evaluation will involve an analysis of the frequency of adverse events and serious adverse events suspected to be related to the treatment. Adverse events will be collected through subject reports or investigator observation. The manifested adverse events will be described narratively with a detailed explanation, and the frequencies of adverse events with and without a relationship to the clinical trial intervention will be recorded and presented using descriptive statistics. Additionally, the number of events per visit between groups will be compared using a Fisher's exact test. For each laboratory test variable, a paired t-test will be used to analyze whether there is a difference between the pre- and post-treatment values.

Schedule Summary

<table>
<tr><th colspan="2"></th><th>Visit 1
(Screening[1])</th><th>Visit 2
(Baseline)</th><th>Visit 3</th></tr>
<tr><th colspan="2">Visit window</th><th>Day -1~0</th><th>Day 0</th><th>Day 7-10</th></tr>
<tr><td colspan="2">Written Consent</td><td>X</td><td></td><td></td></tr>
<tr><td colspan="2">Demographic Survey</td><td>X</td><td></td><td></td></tr>
<tr><td colspan="2">Medical History</td><td>X</td><td></td><td></td></tr>
<tr><td colspan="2">Physical Exam</td><td>X</td><td></td><td></td></tr>
<tr><td colspan="2">Vital Signs</td><td>X</td><td>X</td><td>X</td></tr>
<tr><td colspan="2">Physical Measurements</td><td>X</td><td></td><td></td></tr>
<tr><td colspan="2">Laboratory Test [2]</td><td>X</td><td></td><td>X</td></tr>
<tr><td colspan="2">Pregnancy Test[3]</td><td>△</td><td></td><td></td></tr>
<tr><td colspan="2">EKG</td><td>X</td><td></td><td></td></tr>
<tr><td colspan="2">Chest X-ray</td><td>△</td><td></td><td></td></tr>
<tr><td colspan="2">PNS (Paranasal Sinuses) X-ray</td><td>△</td><td></td><td></td></tr>
<tr><td colspan="2">Pattern Identification for Common Cold Questionnaire</td><td>X</td><td></td><td></td></tr>
<tr><td colspan="2">Inclusion and Exclusion Criteria Evaluation</td><td>X</td><td></td><td></td></tr>
<tr><td colspan="2">Random Assignment</td><td></td><td>X</td><td></td></tr>
<tr><td colspan="2">Administration of Investigational Product</td><td></td><td colspan="2">Daily</td></tr>
<tr><td rowspan="11">Assessments</td><td rowspan="2">Nasal Congestion Severity Score</td><td rowspan="2">X</td><td colspan="2">Daily</td></tr>
<tr><td>X</td><td>X</td></tr>
<tr><td rowspan="2">WURSS-21-K</td><td rowspan="2"></td><td colspan="2">Daily</td></tr>
<tr><td>X</td><td>X</td></tr>
<tr><td rowspan="2">Nasal Symptom Score VAS</td><td rowspan="2"></td><td colspan="2">Daily</td></tr>
<tr><td>X</td><td>X</td></tr>
<tr><td rowspan="2">Systemic Symptom Score VAS</td><td rowspan="2"></td><td colspan="2">Daily</td></tr>
<tr><td>X</td><td>X</td></tr>
<tr><td rowspan="2">Duration of Common Cold</td><td rowspan="2"></td><td colspan="2">Daily</td></tr>
<tr><td></td><td>X</td></tr>
<tr><td>Recovery of Common Cold</td><td></td><td></td><td>X</td></tr>
</table>

X: All participants, △: If considered necessary by the investigator

[1] Screening test should be conducted within one day before Visit 2 and the results of clinical laboratory test should be able to be checked at Visit 2.

[2] CBC (erythrocyte count, leukocyte count, hemoglobin, and hematocrit); LFT (AST, ALT, BUN, creatinine, and glucose).

[3] If pregnancy status is uncertain, urine hCG test for pregnancy confirmation following will be conducted, and the result must be negative.

	Global Evaluation of Efficacy			X
	EQ-5D-5L		X	X
Prescription and Delivery of Assigned Investigational Product			X	
Distribution and Instruction for Common Cold Dairy			X	
Compliance Test				X
Common Cold Diary Check				X
Combined Medication Evaluation			X	X
Dropout Status Check			X	X
Adverse Reaction Evaluation			X	X
Instruction for Visit		X	X	
Completion of Trial Check				X

1. Title and Phase of the Clinical Trial

1.1. Title of the Clinical Trial

Efficacy and safety of Galgeun-tang-ga-cheongung-sinyi for nasal congestion with common cold: a randomized, double-blind, placebo-controlled, parallel, multicenter clinical trial

(Protocol No. KIOM_2024_CC_GGTCS)

1.2. Phase of the Clinical Trial

Investigator-Initiated Trial

2. Clinical Trials Institutions, Central Laboratory, Principal Investigators

2.1. Names and Addresses of Clinical Trials Institutions

Daejeon University Daejeon Korean Medicine Hospital/75, Daedeok-daero 176beon-gil, Seo-gu, Daejeon, South Korea
Kyung Hee University Korean Medicine Hospital/23, Kyungheedae-ro, Dongdaemun-gu, Seoul, South Korea
Pusan National University Korean Medicine Hospital/20, Geumo-ro, Mulgeum-eup, Yangsan-si, Gyeongsangnam-do, South Korea

2.2. Principal Investigators

Yang-Chun Park/Daejeon University Daejeon Korean Medicine Hospital
Beom-Joon Lee/Kyung Hee University Korean Medicine Hospital
Jun-Yong Choi/Pusan National University Korean Medicine Hospital

2.3. Monitoring

Korea Institute of Oriental Medicine Seo-Bok Nam CRA
Korea Institute of Oriental Medicine Ae-Ran Kim CRA
1672, Yuseong-daero, Yuseong-gu, Daejeon, South Korea

3. Background

Common cold is an upper respiratory tract infection syndrome caused by a mild virus. It occurs 2-4 times a year in adults and 6-8 times in children, causing personal distress and economic burden by leading to absenteeism from work and school[1]. According to statistical data from the Health Insurance Review and Assessment Service, the total number of annual outpatient visits for common cold (J00) in 2023 was approximately 4.57 million, with total medical care benefit costs reaching about 174.9 billion Korean won. Common cold ranks 13th among the most frequent outpatient diagnoses in western medicine and 14th in Korean medicine[2].

Symptoms of the common cold vary, including rhinorrhea, nasal congestion, cough, headache, sore throat, myalgia, and fever, which interfere with daily life and reduce an individual's work capacity[3]. While common cold is generally a self-limiting disease confined to the upper respiratory tract, it can occasionally spread to adjacent organs, causing other symptoms, or, rarely, lead to complications from bacterial infections[4]. Although symptomatic treatment is typically employed for the common cold, some medications used in clinical practice have limitations. For example, first-generation antihistamines can cause severe drowsiness, and non-steroidal anti-inflammatory drugs (NSAIDs) can cause gastrointestinal issues, making long-term use difficult[5]. Therefore, there is a need for safer and more effective treatments for the common cold, and Korean medicine treatments can be a viable option.

Rhinovirus is the most common viral cause of the common cold, accounting for 30-50% of cases. The most frequent symptoms are rhinorrhea, nasal congestion, and sneezing, with other symptoms including sore throat, headache, cough, and fever. Thus, prescriptions for nasal symptoms are frequently used in clinical practice[4]. However, existing standard clinical practice guidelines for the common cold in Korean medicine have confirmed evidence for herbal treatments for symptoms such as cough, rhinorrhea, fever, and systemic pain, but lack sufficient evidence for nasal congestion. Therefore, there is a need to create new evidence in this area[6].

Galgeun-tang-ga-cheongung-sinyi (GGTCS) is an herbal formula composed of Puerariae Radix, Ephedrae Herba, Cinnamomi Ramulus, Paeoniae Radix, Glycyrrhizae Radix et Rhizoma, Zingiberis Rhizoma, Zizyphi Fructus, Cnidii Rhizoma, and Magnoliae Flos. It is a modification of the Galgeun-tang from the Shanghan Lun (Treatise on Cold Damage), with the addition of Cnidii Rhizoma and Magnoliae Flos. Galgeun-tang has been reported to activate immune cells, suppress allergic reactions, and possess anti-inflammatory, antipyretic, and analgesic effects[78]. A study analyzing data from the Health Insurance Review and Assessment Service found that Galgeun-tang was the fourth most frequently used herbal insurance preparation prescribed to patients with allergic rhinitis[9]. GGTCS adds Cnidii Rhizoma, which invigorates blood and Qi, dispels wind, and relieves pain, and Magnoliae Flos, which disperses wind-cold and unblocks the nasal passages, to the original formula[10]. A preceding study reported an immediate therapeutic effect of GGTCS in patients with allergic nasal congestion as their main complaint[11]. Thus, GGTCS is expected to be effective for common cold patients with nasal congestion as their primary symptom.

4. Objectives

To evaluate and compare the efficacy and safety of GGTCS compared with placebo for nasal congestion with common cold.

5. Participants

5.1. Inclusion Criteria

1) Men and women aged between 19 and 75 years
2) Diagnosed with the common cold, symptoms occurring within 72 hours of the start of the trial
3) More than two symptoms related to the common cold including runny nose, nasal congestion, sneezing, sore throat, cough, headache, chills, and body aches
4) Nasal congestion severity score greater than 2
5) Those who voluntarily decide to participate in this clinical trial and give written informed consent to abide by the precautions after listening a detailed explanation of this clinical trial and fully understanding it.

5.2. Exclusion Criteria

1) Those with sinusitis, allergic rhinitis, pneumonia, influenza, COVID-19, cough or sore throat with sudden fever above 38 ℃, bronchitis, otitis media, tonsillitis (if accurate examination is necessary, paranasal sinuses (PNS) view or chest X-ray will be conducted)
2) Diagnosis of chronic respiratory disease (chronic obstructive pulmonary disease, bronchial asthma, bronchiectasis, interstitial lung disease, and other chronic respiratory diseases)
3) Those who had taken antibiotics, antivirals, steroids, nasal decongestants, antihistamines, antitussives, expectorants, or other medications to alleviate symptoms of the common cold within 1 week of screening
4) Liver or renal impairment (ALT, AST, and creatinine ≥ 3 times the upper normal limit at screening)
5) Those with generic disorders such as galactose intolerance, Lapp lactase deficiency, or glucose-galactose malabsorption
6) Pseudo-aldosteronism or myopathy induced by hypokalemia
7) Comorbidities that interrupt the treatment of cancers or clinically significant disorders of the kidney, liver, psychiatric system, cardiovascular system, respiratory system, endocrine system, or central nervous system thus interfering with the assessment of efficacy and safety of the investigational drug or completion of the clinical study
8) Unregulated hypertension (systolic blood pressure ≥ 160 mmHg or diastolic blood pressure ≥100 mmHg)
9) Unregulated diabetes mellitus (fasting blood sugar ≥ 180 mg/dL)
10) History of hypersensitivity reaction to materials of drugs used in this clinical trial
11) History of alcoholism or substance abuse
12) Current smokers or those with history of smoking more than 30 packs/year
13) Pregnant or lactating women
14) Those who do not use medically acceptable contraception (e.g., intrauterine device with proven pregnancy failure rate in spouse or partner, simultaneous use of barrier method for men or

women with spermicide, or surgical procedures, such as vasectomy, tubectomy, tubal ligation, or hysterectomy for oneself or one's partner) during the clinical trial

15) Those who participated in other clinical trials within 30 days before participation in this clinical trial
16) Those determined by investigators to be ineligible to participate in this trial

5.3. Sample Size

1) Sample Size

	GGTCS group	Placebo group	Total
Final efficacy population (PP)	70	70	140
Population considering dropout rate (20%)	88	88	176

2) Rationale

This clinical trial is designed to evaluate the efficacy and safety of GGTCS for patients with nasal congestion due to the common cold. The primary outcome measure is the WRUSS-21-K. The change in the WRUSS-21-K from baseline to Day 7 (after a total of 21 doses, 3 times a day for 7 days) will be compared between the test group (GGTCS) and the control group (placebo).

The hypothesis for this clinical trial is as follows:

[Hypothesis] H_0(Null Hypothesis): $\mu_t = \mu_c$ vs. H_1(Alternative Hypothesis): $\mu_t \neq \mu_c$

μ_t : The mean change in the WRUSS-21-K score from baseline to Day 7 in the test group.
μ_c : The mean change in the WRUSS-21-K score from baseline to Day 7 in the control group.

Table 1. Change in WRUSS-21-K of Eungyo-san, Samso-eum, placebo between pre- and post-treatment.

	Pre-Treatment	Post-Treatment	Change (Before-After)*
Eumgyo-san (n=44)	56.28(30.47)	13.86(22.43)	44.00(22.27)
Samso-eum (n=42)	54.74(30.98)	17.05(24.29)	40.12(18.78)
Placebo (n=42)	66.48(31.64)	33.33(30.02)	29.09(21.89)

*: Change adjusting WRUSS-21-K score of baseline.

Previous studies (K.-I. Kim, M. Hong) showed that the mean (standard deviation) change in the WRUSS-21-K score from pre- to post-treatment was 29.09 (21.89) for the placebo group, 44.00 (22.27) for the Eungyo-san treatment group, and 40.12 (18.78) for the Samso-eum treatment group. The mean difference in effect was 14.91 for the Eungyo-san treatment group compared with the placebo group, with a pooled standard deviation of 22.09. The mean difference in effect for Samso-eum compared with the placebo group was 11.03, with a pooled standard deviation of 20.39. In this clinical trial, the significance level (α) was set at a two-sided 5%, and the power (1-β) is 80%. Based on the results of the previous studies, the effect difference is set at 10 and the standard deviation at 21. The calculated number of subjects required to confirm the effect of the GGTCS group compared with the placebo group is as follows.

$$\left\{\frac{2\left(z_{1-\frac{\alpha}{2}}+z_{\beta}\right)^{2}*\sigma^{2}}{|\mu_T-\mu_c|}\right\}=\left\{\frac{2(1.96+0.84)^2*21^2}{10^2}\right\}=69.227\approx 70$$

Based on the 1:1 allocation ratio between the treatment and control groups, the required number of subjects per group is 70. Considering a 20% dropout rate for this clinical trial, a total of 176 subjects (88 per group) are needed.

Subjects will be recruited from multiple centers, with a target of 88 subjects from Daejeon University Daejeon Korean Medicine Hospital, and 44 subjects each from Kyung Hee University Korean Medicine Hospital and Pusan National University Korean Medicine Hospital. However, the number of subjects recruited per center may vary depending on the recruitment speed. The final allocation of subjects per center will be re-evaluated by the Central Data Management (DM) team at the midpoint (50%) of the total study period, based on the recruitment speed of each center. If the number of subjects per center changes, an independent statistician will generate new codes as needed, considering the block size, and these will be reflected in the randomization schedule for each center. Investigational products will be provided to each study site based on their initial number of subjects. Additional products for sites with increased subject numbers will be provided by the drug manufacturer (National Institute of the Korean Medicine Development) according to the newly generated randomization codes. Sites with a reduced subject number will be instructed to discard the remaining randomization codes.

5.4. Recruitment

To recruit study subjects, regular promotion will be carried out through mass media, such as newspaper flyers and daily newspapers, and promotional materials will be posted on bulletin boards inside and outside the sites. In the event of a delay in subject enrollment during the clinical trial, local advertisements will be implemented, including subway, bus, and apartment bulletin board.

6. Trial Design

6.1. Period of Trial

The duration of this clinical trial will be 24 months from the date of Institutional Review Board (IRB) approval of this protocol. However, it may be extended depending on the subject enrollment speed.

6.2. Procedure

This clinical trial is designed as a randomized, double-blind, placebo-controlled, parallel-group, investigator-initiated trial to evaluate the efficacy and safety of GGTCS in patients with nasal congestion due to the common cold.
After voluntarily signing the informed consent form, subjects will be assessed for eligibility based on the inclusion and exclusion criteria. Only eligible subjects will be randomized to either the test group or the control group in the order of their enrollment. The assigned subjects will take the investigational product (test drug or placebo) for a maximum of 7 days.
The study will investigate the following assessments of the the WURSS-21-K total score, symptom score, quality of life score, Nasal Congestion Severity Score, Nasal Symptom Score VAS, Systemic Symptom Score VAS, and duration of cold symptoms in each time points, Global Evaluation of Efficacy score on Day 7, EQ-5D-5L score on Day 7 from baseline, and the change in WURSS-21-K total score according to the Cold Pattern Identification (Wind-Cold, Wind-Heat, or other types).

- Symptom Improvement Effect
- Quality of Life Assessment
- Comparison of effects based on Korean medicine diagnostic criteria

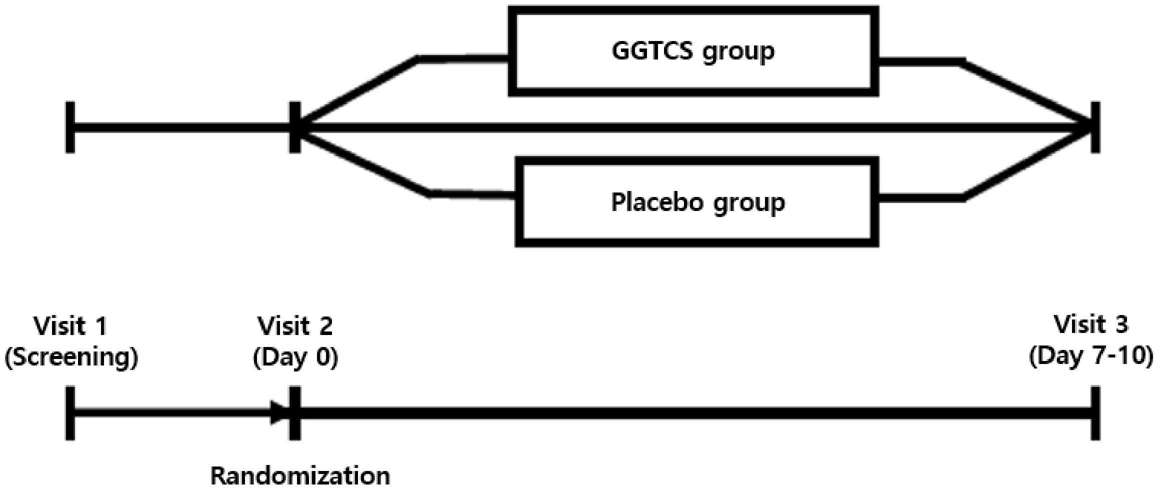

<Flow Diagram of Clinical Trial>

6.3. Randomization

- This clinical trial will use blocked randomization, without stratification.
- A statistician or an independent researcher will use the statistical program SAS® Version 9.4 (SAS Institute Inc., Cary, NC, USA) to generate a predetermined randomization list based on a table of random numbers, and will assign subjects to either the test group or the control group. This list will then be provided to the randomization manager (a pharmaceutical company representative).
- The pharmaceutical company's labeling manager will label the investigational products with unique randomization numbers and deliver them to the institutional pharmacist. To ensure blinding of subjects, investigators, monitors, and pharmacists, the test and placebo drugs will be kept identical in appearance, with only the randomization number on the label. The randomization manager will be an individual independent of the study interventions and assessments, and will not disclose the information until the completion of the clinical trial, unless there are special circumstances.
- The identification codes for each subject on the randomization list will be sealed in a small envelope in advance. The labels will not show any difference between the groups. The allocation list of unique codes will be managed by the unblinding manager of the clinical trial, and will not be disclosed until the study is fully completed, except in cases where access to the code is unavoidable due to a serious adverse event.
- Subjects who meet all inclusion and exclusion criteria will be assigned to either the test group or the control group according to the pre-generated randomization list, starting from the lowest available number. The institutional pharmacist will sequentially supply the investigational products to the subjects based on their assigned identification code numbers.
- An independent statistician will generate randomization codes for each site, incorporating the site code. If the number of subjects per site changes, the independent statistician will generate new codes as needed, considering the block size, and these will be reflected in the randomization list for each site.
  (Site codes: Daejeon University Daejeon Korean Medicine Hospital 01, Kyung Hee University Korean Medicine Hospital 02, Pusan National University Korean Medicine Hospital 03)

6.4. Blinding

1) Blinding for Subjects
   - Subjects will be aware only that they are receiving a different drug (either the test drug or the control drug) but will not be exposed to information about which one is the test drug.
   - The subject identification numbers will be developed to prevent differentiation between the test group and the control group.
   - The test drug and the control drug have been developed to be identical in size, appearance, and shape, and are packaged in identical wrapping.

2) Blinding for Investigators

- Investigators will oversee the progress of the study but will not be involved in the randomization process and will remain blinded to the type of treatment each subject receives.

3) Blinding for the Randomization Manager
    - Based on the randomization results, the randomization manager will label the test and control drugs and provide them to the institutional pharmacist. The randomization results will be stored in an opaque envelope within a double-locked cabinet. The randomization results cannot be accessed until the end of the clinical trial, unless there are special circumstances.

4) Unblinding Procedure
    - During the clinical trial, if a serious adverse event or other emergency situation occurs and it is absolutely necessary to protect the rights and safety of the subject, the unblinding procedure may be performed at the investigator's request. Unblinding should be considered on a case-by-case basis and only in a serious medical emergency. Furthermore, unblinding should only occur if knowledge of the treatment arm will affect the patient's care. If the principal investigator determines that unblinding is absolutely necessary, they will perform the unblinding and notify the monitor of the facts via verbal communication or in writing (e-mail). In such cases, the investigator must document the reason and facts of the unblinding within 24 hours.

5) Emergency Envelopes
    - Since most participants in this study will be blinded, emergency envelopes containing allocation information will be prepared for unforeseen circumstances. These envelopes will be opaque and stored in a secure and accessible location. The reason for opening an emergency envelope must be documented.

7. Dropout and Early Termination Criteria

7.1. Dropout

Dropout refers to the premature termination of a subject's participation before completing all study procedures. The investigator may stop the treatment and observation and withdraw the subject, and the subject may voluntarily withdraw from the study at any time. A subject who meets any of the following criteria may be withdrawn from the study at the discretion of the principal investigator or responsible investigator. In the event of any of the following, the administration of the study drug and the trial itself may be discontinued, leading to withdrawal.

1) If a subject is confirmed to violate the inclusion/exclusion criteria after screening.
2) If a systemic disease, not discovered during pre-administration screening, is found in the subject.

3) If the investigator determines that continuous administration is inappropriate due to a serious adverse event in the subject.
4) If the investigator determines that continuous administration is inappropriate due to the severity of an adverse event.
   - Subjects who discontinue early due to an adverse event may receive appropriate treatment for the event if necessary.
5) If the investigator determines that the subject's symptoms have worsened and other treatment is required.
6) If the subject or their legal representative withdraws consent for participation in the clinical trial or requests to stop the administration of the investigational product (due to unsatisfactory therapeutic effect during the trial period).
7) If the subject's visits and follow-up are not possible.
8) If the subject violates the clinical trial protocol or leaves the hospital without permission, making it difficult to continue the trial.
9) If the subject takes any prescribed medication or over-the-counter medication that could affect the study's outcome (e.g., safety, pharmacokinetic properties) without the supervising physician's instruction during the clinical trial period (all periods after screening).
10) If the subject's medication compliance is significantly low, at less than 70%.
11) Other cases where the investigator judges the continuation of the clinical trial to be inappropriate.

The date, reason, and a summary of the dropout will be recorded on the Case Report Form (CRF) conclusion page. The investigator should make every effort to follow up with subjects who have dropped out or withdrawn from the trial. Subjects are not obligated to state the reason for their withdrawal. However, the investigator must record the reason for discontinuation or the failure to ascertain the reason in the CRF. For subjects who discontinue due to an adverse event, follow-up examinations will be conducted as deemed necessary by the investigator.

Withdrawn and dropped subjects will not be replaced with new subjects.

7.2. Early Termination

The principal investigator may prematurely terminate this clinical trial after discussion with the funding organization if any of the following situations, which require the discontinuation of the study, occur. If a subject is withdrawn from the study before drug administration, they will be considered a screening failure, and it is not mandatory to complete all assessments.

The criteria for early discontinuation of the clinical trial are as follows:

1) If an unexpected or unacceptable risk is discovered in the subjects.

2) If moderate or severe adverse events, judged to be related to the investigational product, occur in more than 25% of all subjects.
   - The severity of adverse events will be evaluated with reference to the National Cancer Institute (NCI) "Common Terminology Criteria for Adverse Events (CTCAE) version 4.0," and a decision will be made on whether to terminate the entire clinical trial.
   - If the study is judged to pose a risk to subjects based on the progression of the adverse event and the causal relationship with the study drug, the investigator will permanently discontinue the subject's treatment in this clinical trial.
   - For subjects who discontinue early due to an adverse event, the investigator or their designee will continue to evaluate the subject until the adverse event is resolved or judged to be permanent.
3) If all subjects have not been enrolled despite a sufficient recruitment period.

7.3. Completion Criteria

The clinical trial will be finally terminated when the planned number of subjects has been enrolled and the integrity of the collected data has been secured.
When the last subject has completed all planned visits and follow-up as per the protocol, the Principal Investigator will record the overall study completion date, compile the data, and perform the efficacy evaluation.
A subject's individual trial completion is defined as having completed all planned visits and assessments as specified in the clinical trial protocol without discontinuation or withdrawal, and the trial is considered complete for that subject.

8. Investigational Product

8.1. GGTCS

Name	Kyungbang Galgeun-tang-ga-cheongung-sinyi
Formulation and Appearance	Light brown granules
Ingredients and Content	Per single dose (3.0g): Puerariae Radix 2.67g, Ephedrae Herba 1.33g, Cinnamomi Ramulus 1.00g, Paeoniae Radix 1.00g, Glycyrrhizae Radix et Rhizoma 0.67g, Zingiberis Rhizoma Crudus 0.33g, Zizyphi Fructus 1.33g, Cnidii Rhizoma 1.00g, Magnoliae Flos 1.00g
Effects	Nasal congestion, sinusitis, chronic rhinitis
Route of Administration	Oral administration
Dosage	Adults: 1 packet (single dose) 3 times a day, before or between meals

Storage Method	Airtight container, room temperature (1-30℃)
Shelf Life	36 months from the date of manufacture
Manufacturer	Kyungbang Pharm Co.

8.2. Placebo

Name	Placebo for Galgeun-tang-ga-cheongung-sinyi
Formulation and Appearance	Light brown granules
Ingredients and Content	Placebo for Kyungbang Galgeun-tang-ga-cheongung-sinyi, containing excipients and colorants, excluding the active ingredients.
Route of Administration	Oral administration
Dosage	Adults: 1 packet (single dose) 3 times a day, before or between meals
Storage Method	Airtight container, room temperature (1-30℃)
Shelf Life	36 months from the date of manufacture
Manufacturer	National Institute of the Korean Medicine Development

8.3. Management of Investigational Product

1) Labeling and Packaging of Investigational Products
    - The study site will receive a sufficient quantity of investigational products from the pharmaceutical company to conduct the clinical trial.
    - The sponsor must provide the labels for the investigational products to the institutional pharmacist in accordance with the regulations specified in the guidelines on clinical trial management. The manufacturing, packaging, and labeling of investigational products must be conducted in accordance with the Good Manufacturing Practice for Investigational Products.
    - The following investigational product label will be applied in accordance with the Manufacturing of Investigational Products

1. The words "For Clinical Trial"<br>2. Name or identification mark of the investigational product<br>3. Number or code number to identify the contents and packaging<br>4. Name, address, and telephone number of the person who received the clinical trial approval<br>5. Expiration date<br>6. Storage conditions<br>7. "Keep out of reach of children."<br>8. Reference code to identify the clinical trial<br>9. Subject identification number, visit number<br>10. Directions for use, if the Principal Investigator deems it necessary (However, instructions may refer to a package insert or other documents)

- Investigational products will be produced and packaged at KGMP-compliant facilities by the manufacturer, the National Institute of the Korean Medicine Development, and supplied to the clinical trial sites. During packaging, each group will be packaged sequentially to prevent mixing, and they will be stored separately by group until labeling. To maintain double-blinding, the test drug and the placebo will be packaged in identical containers with the same quantity. The investigational product will be individually packaged for each subject, and each single dose will be individually packaged for accurate administration. The supply provided to each subject will contain a 7-day supply (3 doses x 7 days = 21 packets).
- Each individual dose packet will be labeled. If the surface area of the individual packet is too small to include all items, a label containing only the following information will be attached to the primary packaging, and all items must be included on the secondary packaging:

1. The words "For Clinical Trial"<br>2. Name or identification mark of the investigational product<br>3. Number or code number to identify the contents and packaging<br>4. Name of the person who received the clinical trial approval<br>5. Expiration date<br>6. Reference code to identify the clinical trial<br>7. Subject identification number

2) Inventory Management of Investigational Products
- The principal investigator must ensure that the investigational products are correctly received by the pharmacist and stored in a secure location, and is responsible for preserving the products from damage before dispensing. The investigational products used in this study will not be used for any purpose other than the clinical trial.
- The principal investigator and the institutional pharmacist at the respective clinical trial site are responsible for the management of the investigational products.

- Investigational products will be stored separately in a locked storage area within the clinical pharmacy. During the pharmacist's absence, the storage area will remain locked to restrict access by individuals not involved in this clinical trial. However, if it is necessary for a subject to receive the investigational product or for a remaining product to be returned to the pharmacy in the pharmacist's unavoidable absence, a delegated investigator (with a medical license) may perform these duties.
- The investigational products must be stored in the clinical pharmacy of the study site in accordance with the clinical trial protocol, Investigator's Brochure (IB), and the Clinical Trial Management Guidelines. The pharmacist must check the storage conditions, expiration date, quantity, and storage status of the investigational products, and must record the measured temperature on a temperature log, which will be kept for record-keeping.
- When dispensing the investigational products to subjects, the pharmacist will complete the product inventory log and the subject-specific dispensing log. If any investigational product is returned by a subject, the details will be recorded in the subject-specific dispensing log.

8.4. Return and Disposal

1) In the event of a clinical trial being suspended or terminated, or if the investigator fails to conduct the trial in accordance with the protocol, unused investigational products will be recalled and disposed of.
   - Criteria for return and disposal:
   - When the expiration date of the investigational product has passed.
   - Unused investigational products after the clinical trial is completed.
   - When there are abnormalities in the contents or packaging.

2) Return of Unused Investigational Products
   - When a subject wishes to return unused investigational products, the quantity and condition of the returned products will be verified and handed over to the pharmacist. The pharmacist will record the details in the subject-specific dispensing log.

3) Disposal of Investigational Products after Trial Completion
   - Unused investigational products will be recalled by the manufacturer and disposed of according to the manufacturer's internal regulations and disposal guidelines.
   - The institutional pharmacist, in consultation with the Principal Investigator, must return the unused investigational products and keep the return receipt and relevant disposal confirmation documents.
   - However, if disposal through the manufacturer is not possible due to their circumstances, the products will be disposed of by the institution according to the drug disposal guidelines of the on-site hospital pharmacy. All records regarding the return and disposal must be created and retained.

9. Methods and Administration

9.1. Investigational Product Administration

The investigational product will be administered once per packet (1 pack) three times a day, before or between meals. The total administration period is a maximum of 7 days (21 packets). The daily dose for each group is as follows. If the cold symptoms disappear before the maximum 7-day administration period, subjects will stop taking the medication and return the remaining investigational products.

- ✓ Test Group (GGTCS): 1 packet (3g) per dose, 3 times/day, for a maximum of 7 days.
- ✓ Control Group (Placebo): 1 packet (3g) per dose, 3 times/day, for a maximum of 7 days.

9.2. Concomitant and Prohibited Medications

1) Permissible Concomitant Medications
    - Medications that subjects have been taking for 4 weeks prior to participating in the clinical trial and are not classified as prohibited drugs may be permitted at the investigator's discretion.
    - Medications used for the transient treatment of other diseases will be co-administered after consultation with the investigator.
    - Detailed information about all concomitant medications (including those for other diseases or for treating adverse events) will be recorded in the subject's Case Report Form (CRF), including the drug name, purpose of administration, dose, and duration.

2) Prohibited Concomitant Medications
    - During the clinical trial period, the use of the following medications that may affect symptoms is prohibited. If the use of a prohibited medication is necessary, the subject must discontinue the clinical trial.
        - ① Antibiotics, antiviral agents, steroids, decongestants, antihistamines, antitussives/expectorants, and other medications expected to relieve nasal congestion.
        - ② Medications administered for the same therapeutic purpose as the investigational product or those that may affect the improvement of nasal congestion symptoms.
        - ③ Medications containing the same active ingredients (Puerariae Radix, Ephedrae Herba, Zizyphi Fructus, Cinnamomi Ramulus, Paeoniae Radix, Glycyrrhizae Radix et Rhizoma, Zingiberis Rhizoma Crudus, Cnidii Rhizoma, and Magnoliae Flos), even if they are not used for the same therapeutic purpose as the investigational product.

9.3. Rescue Medication

Subjects are permitted to take Acetaminophen if they experience fever (38°C or higher) or extreme pain. However, the maximum dose is limited to 6 tablets/day and 18 tablets/week (500mg/tablet). Subjects who complain of a high fever of 39°C or higher will be withdrawn from the study.

9.4. Compliance Assessment

To assess medication compliance with the investigational product, the number of doses taken and returned will be checked. Subjects should bring the empty packets and any unconsumed medication from the previous visit, along with their completed cold diary. The investigator will confirm whether the subject took the provided medication and will instruct them to return any remaining medication at the visit, and to avoid overdosing or losing the product. Medication compliance (%) will be calculated as shown below, recorded in the CRF to one decimal place. Compliance of 70% or more is considered "acceptable".

$$\text{Compliance (\%)} = \frac{\text{Number of investigational products actually taken}}{\text{Number of investigational products that should have been taken}} \times 100$$

When calculating the number of investigational products that should have been taken, the rule is to start taking the medication on the evening of the prescription date and to continue taking it until the evening before the next visit. The compliance will be calculated based on the amount that should have been taken and the amount actually taken from the evening of the prescription date until the evening before the next visit. If the subject stops taking the medication due to symptom resolution, compliance will be calculated based on the amount taken from the evening of the prescription date until the evening of the day before the symptoms resolved.
Subjects with a final medication compliance of less than 70% will be excluded from the PP analysis.

10. Procedure

10.1. Timetable

	Visit 1 (Screening[1])	Visit 2 (Baseline)	Visit 3
**Visit window**	**Day -1~0**	**Day 0**	**Day 7-10**
Written Consent	X		
Demographic Survey	X		

X: All participants, △: If considered necessary by the investigator

[1] Screening test should be conducted within one day before Visit 2 and the results of clinical laboratory test should be able to be checked at Visit 2.

Medical History		X		
Physical Exam		X		
Vital Signs		X	X	X
Physical Measurements		X		
Laboratory Test [1]		X		X
Pregnancy Test[2]		△		
EKG		X		
Chest X-ray		△		
PNS (Paranasal Sinuses) X-ray		△		
Pattern Identification for Common Cold Questionnaire		X		
Inclusion and Exclusion Criteria Evaluation		X		
Random Assignment			X	
Administration of Investigational Product			Daily	
Assessments	Nasal Congestion Severity Score	X	Daily	
			X	X
	WURSS-21-K		Daily	
			X	X
	Nasal Symptom Score VAS		Daily	
			X	X
	Systemic Symptom Score VAS		Daily	
			X	X
	Duration of Common Cold		Daily	
				X
	Recovery of Common Cold			X
	Global Evaluation of Efficacy			X
	EQ-5D-5L		X	X
Prescription and Delivery of Assigned Investigational Product			X	
Distribution and Instruction for Common Cold Dairy			X	
Compliance Test				X
Common Cold Diary Check				X
Combined Medication Evaluation			X	X

1 CBC (erythrocyte count, leukocyte count, hemoglobin, and hematocrit); LFT (AST, ALT, BUN, creatinine, and glucose).

2 If pregnancy status is uncertain, urine hCG test for pregnancy confirmation following will be conducted, and the result must be negative.

Dropout Status Check		X	X
Adverse Reaction Evaluation		X	X
Instruction for Visit	X	X	
Completion of Trial Check			X

10.2. Observation and Evaluation by Visit Schedule

10.2.1. Visit 1 (Screening Visit/Day -1 ~ Day 0)

1) Obtaining Written Consent
   - Before conducting any clinical trial procedures, the investigator must use the 'Informed Consent Form for Subjects' to explain the study title, purpose, duration, methods, expected benefits, risks, subject protection, and the right to voluntarily consent or withdraw. Sufficient time will be given for the subject to make a voluntary decision. Once the subject decides to participate, they will be asked to sign the form. A copy will be provided to the subject, and the original will be retained by the study site.
   - A screening number will be assigned in the order that consent is obtained.

     ✓ KIOM_2024_CC_GGTCS_0X_SOOO

2) Demographic Survey
   - The subject's initials (in English), sex, date of birth, age, history of alcohol use (whether they drink, amount of alcohol consumption), and smoking history (whether they smoke, amount of smoking) will be surveyed and recorded in the Case Report Form (CRF).

3) Medical and Treatment History (Drug History) Survey
   - The subject's medical history and drug administration history will be investigated in detail through interviews and a review of past medical records. The presence of a history of sinusitis, allergic rhinitis, pneumonia, influenza, COVID-19, bronchitis, otitis media, tonsillitis, chronic respiratory diseases (COPD, bronchial asthma, bronchiectasis, interstitial lung disease, other chronic respiratory diseases), malignant tumors, active infections, blood coagulation disorders, neuropsychiatric, hepatic, renal, cardiovascular, respiratory, endocrine, or central nervous system diseases will be checked. If a history of these diseases is present, the subject will be excluded.
   - A medical history from within the past 3 years, including the diagnosis name, date of diagnosis, and whether it is ongoing at the time of screening, will be recorded in the CRF. Surgical history from within the past 3 years, including the name of the surgery and date, will also be recorded.

- Drug history will be investigated for medications taken within the last 3 months and those currently being taken at the time of screening. For prior/concomitant medications, the drug name (brand name), total daily dose, route of administration, start date, end date, whether it is ongoing, and purpose will be recorded.

4) Physical Examination
   - Through a review of medical records and an interview, the investigator will examine the cardiovascular, peripheral vascular, skin/mucosa, eyes (excluding vision impairment), ENT, respiratory, musculoskeletal, endocrine, renal/genitourinary, neurological/psychiatric, oncological, infectious disease, and gastrointestinal/hepatic/biliary systems, as well as allergies and drug hypersensitivity reactions, and record whether they are normal or abnormal. If abnormal, a medical opinion will be recorded in the CRF, and suitability for participation in the clinical trial will be confirmed.

5) Vital Signs Measurement
   - Vital signs including blood pressure (systolic, diastolic), pulse rate, and body temperature will be measured. Blood pressure and pulse rate will be measured using an automated sphygmomanometer while the subject is seated after resting for at least 3 minutes at every visit. Body temperature will be measured using an electronic thermometer after blood pressure is measured.

6) Physical Measurements
   - The subject's height and weight will be measured and recorded via a body composition analysis.

7) Laboratory and Pregnancy Test
   - The laboratory test items are as follows. For women of childbearing potential, a urine hCG test will be measured after an interview if there is any uncertainty about pregnancy status.
     - ✓ Complete blood count: RBC, WBC, Hemoglobin, Hematocrit, Platelet
     - ✓ Liver function test: AST, ALT, BUN, Creatinine, Glucose
   - The investigator may conduct additional tests related to any abnormal results.

8) EKG
   - EKG will be performed to check for abnormal findings, and if any are present, the subject will be excluded. EKG results from within 2 weeks prior to obtaining consent are acceptable as screening results (when using test results from another institution, a results sheet that can confirm the findings must be secured.).
   - Investigator may conduct additional tests related to any abnormal results.

9) Chest, PNS (paranasal sinuses) X-ray

- Chest or PNS X-ray will be performed if the investigator deems a differential diagnosis necessary.
- Chest X-ray will be checked for findings suggestive of diseases that are part of the exclusion criteria, such as consolidation, atelectasis, emphysema, fibrosis, or a mass.
- PNS X-ray will be checked for findings suggestive of sinusitis, such as mucoperiosteal thickening, haziness, or air-fluid levels in the sinuses.

10) Nasal Congestion Severity Score
- This is a 0-3 point scale used to assess the severity of nasal congestion symptoms, which subjects will self-evaluate (0=none, 1=mild, 2=moderate, 3=severe)[12].
- It will be assessed at Visit 1.

11) Pattern Identification for Common Cold Questionnaire
- It will be used to classify the subject's pattern type to see if there is an effect difference by pattern.
- It classifies the patterns as Wind-Cold type, Wind-Heat type, or other types.
- It will be assessed at Visit 1.

12) Confirmation of Inclusion/Exclusion Criteria
- Only for subjects who have voluntarily signed the clinical trial consent form, final confirmation of eligibility based on the inclusion and exclusion criteria will be made at the screening visit using the demographic survey, medical and treatment history (drug history) survey, physical examination, vital signs measurement, laboratory tests, pregnancy test, Chest and PNS X-rays, and EKG. The final decision on suitability will be made by referencing the laboratory test results and recorded. The subject will then be notified by phone to inform them of the clinical trial schedule and precautions, and to set the date for the baseline visit.

10.2.2. Visit 2 (Baseline/Day 0)

A randomization number will be assigned within one day after the screening visit (subject eligibility will be notified by phone), and the following assessments will be performed.

1) Confirmation of Concomitant Medications
- Concomitant medications and any changes since the last visit will be confirmed.

2) Confirmation of Adverse Events
- The investigator must record all adverse events that occur during the clinical trial. The investigator will assess the adverse event and record the name of the event, onset date, resolution date, severity, causality, actions taken, and outcome.

3) Vital Signs Measurement

- Vital signs including blood pressure (systolic, diastolic), pulse rate, and body temperature will be measured. Blood pressure and pulse rate will be measured using an automated sphygmomanometer while the subject is seated after resting for at least 3 minutes at every visit. Body temperature will be measured using an electronic thermometer after blood pressure is measured.

4) Assessments of WURSS-21-K, Nasal Symptom Score VAS, Systemic Symptom Score VAS, EQ-5D-5L

5) Assignment of Randomization Number

- Subjects who are deemed eligible for this clinical trial based on the inclusion/exclusion criteria will be assigned a randomization number in the following format, in the order of their enrollment.
- Site codes: Daejeon University Daejeon Korean Medicine Hospital 01, Kyung Hee University Korean Medicine Hospital 02, Pusan National University Korean Medicine Hospital 03

✓ KIOM_2024_CC_GGTCS_0X_ROOO

6) Prescription and Dispensing of Investigational Products

- A 7-day supply of the investigational product will be prescribed and dispensed for consumption until the next visit. Subjects will be instructed on the dosage, administration, precautions, and how to return any remaining investigational products to prevent loss or overdose. They will also be encouraged to return empty packets if possible.

7) Distribution and Instruction on Cold Diary

- Subjects will be given a cold diary to be completed until the next visit and will be trained to record their WURSS-21-K, Nasal Congestion Severity Score, Nasal Symptom Score VAS, Systemic Symptom Score VAS, and duration of cold symptoms daily.

8) Instruction on Next Visit Schedule

- Subjects will be instructed on the schedule for their next visit.

10.2.3. Visit 3(Final visit/Day 7-10)

1) Confirmation of Concomitant Medications

- Concomitant medications and any changes since the last visit will be confirmed.

2) Confirmation of Adverse Events

- The investigator must record all adverse events that occur during the clinical trial. The investigator will assess the adverse event and record the name of the event, onset date, resolution date, severity, causality, actions taken, and outcome.

3) Vital Signs Measurement

- Vital signs including blood pressure (systolic, diastolic), pulse rate, and body temperature will be measured. Blood pressure and pulse rate will be measured using an automated sphygmomanometer while the subject is seated after resting for at least 3 minutes at every visit. Body temperature will be measured using an electronic thermometer after blood pressure is measured.

4) Assessments of WURSS-21-K, Nasal Congestion Severity Score, Nasal Symptom Score VAS, Systemic Symptom Score VAS, Global Evaluation of Efficacy, EQ-5D-5L, and duration of cold symptoms.

5) Laboratory Test
    - Subjects will undergo laboratory tests.
        - ✓ Complete blood count: RBC, WBC, Hemoglobin, Hematocrit
        - ✓ Liver function test: AST, ALT, BUN, Creatinine
    - The investigator may conduct additional tests related to any abnormal results.

6) Return of Investigational Products and Compliance Test
    - Subjects must return any investigational products remaining from the last 7 days. The pharmacist will check the quantity of returned products, document the medication administration to confirm that each subject took the correct dose, and verify that the investigational product inventory matches the usage records.

7) Cold Diary Check
    - The cold diary distributed at Visit 2 will be checked to ensure it has been properly completed.

10.2.4. Additional Visits

Additional visits can be made as needed at the subject's request or at the discretion of the investigator. During an additional visit, concomitant medications and adverse events will be checked, vital signs will be measured, and laboratory tests may be conducted at the investigator's discretion.

10.2.5. Monitoring for Symptom Worsening

If a subject's symptoms worsen during a visit or between visits, medical examinations and actions will be

taken. For subjects with a possibility of rapid symptom worsening, their condition will be checked by phone within 3 days after the visit. If a high fever or extreme pain occurs, the use of a rescue medication (Acetaminophen) is permitted. If the investigator determines that a prohibited concomitant medication is necessary, the study will be discontinued for that subject and the required medication will be provided.

10.3. Outcome Measures

10.3.1. Primary Outcome Measures

10.3.1.1. Wisconsin Upper Respiratory Symptom Survey, Korean version(WURSS-21-K) Total Score

- The WURSS-21 is a validated assessment tool for measuring the severity of common cold symptoms and quality of life[13]. A study on the reliability and validity of the Korean version, WURSS-21-K, has been conducted[14].
- It consists of a total of 21 questions: one question about the overall feeling of illness, 10 questions about the severity of cold symptoms, 9 questions about quality of life, and one question to compare symptom improvement/worsening compared to the previous day.
- For the question on symptom improvement/worsening, subjects will choose one of seven options: "very much better," "somewhat better," "a little better," "about the same," "a little worse," "somewhat worse," or "very much worse."
- The other 20 questions are structured on a 0-7 Likert scale (0 = none at all, 1 = very little, 3 = a little, 5 = moderate, 7 = very).
- The total score, which is the sum of the scores for all 21 questions, will be calculated and evaluated.
- At Visit 2, a diary containing the WURSS-21-K will be distributed and subjects will be instructed to complete it daily by themselves. It will be collected at Visit 3.

10.3.2. Secondary Outcome Measures

10.3.2.1. WURSS-21-K Symptom Score

- This score is calculated by summing the scores of the 10 questions in the WURSS-21-K that evaluate symptoms.
- Daily diary containing the WURSS-21-K will be distributed at Visit 2, and subjects will be instructed to complete it daily. It will be collected at Visit 3.

10.3.2.2. WURSS-21-K Quality of Life Score

- This score is calculated by summing the scores of the 9 questions in the WURSS-21-K that evaluate quality of life.
- Daily diary containing the WURSS-21-K will be distributed at Visit 2, and subjects will be instructed to complete it daily. It will be collected at Visit 3.

10.3.2.3. Nasal Congestion Severity Score

위스콘신 상기도 감염 설문 - 21 —일과 증상 기록

날짜(며칠째):	날짜(연월일):	시간: 오전 오후 :	번호:

다음 각각의 항목에 대하여 동그라미안에 체크하세요:

	아프지 않다 0	아주 조금 1	2	조금 3	4	보통 5	6	매우 7
오늘 얼마나 아프다고 느끼십니까?	○	○	○	○	○	○	○	○

각각의 증상에 대해 지난 24시간 동안의 감기증상으로 힘들었던 정도를 평가해주세요:

	이러한 증상은 없었음 0	아주 조금 1	2	조금 3	4	보통 5	6	매우 7
콧물	○	○	○	○	○	○	○	○
코막힘	○	○	○	○	○	○	○	○
재채기	○	○	○	○	○	○	○	○
목이 아픔	○	○	○	○	○	○	○	○
목이 간질거리는 느낌	○	○	○	○	○	○	○	○
기침	○	○	○	○	○	○	○	○
쉰목소리	○	○	○	○	○	○	○	○
머리가 맑지 않고 묵직함	○	○	○	○	○	○	○	○
가슴이 답답함	○	○	○	○	○	○	○	○
피로감	○	○	○	○	○	○	○	○

지난 24시간 동안, 감기가 당신의 삶의 질에 어떠한 영향을 미쳤나요?

	전혀 없었음 0	아주 조금 1	2	조금 3	4	보통 5	6	매우 7
명확하게 생각하는데 지장을 주었다	○	○	○	○	○	○	○	○
잠을 잘 자는데 지장을 주었다	○	○	○	○	○	○	○	○
쉽게 숨을 쉬는데 지장을 주었다	○	○	○	○	○	○	○	○
걷기, 계단 오르기, 운동에 지장을 주었다	○	○	○	○	○	○	○	○
일상적인 활동에 지장을 주었다	○	○	○	○	○	○	○	○
집 밖의 일을 하는데 지장을 주었다	○	○	○	○	○	○	○	○
집안일을 하는데 지장을 주었다	○	○	○	○	○	○	○	○
다른 사람들과 어울리는데 지장을 주었다	○	○	○	○	○	○	○	○
개인적인 생활을 하는데 지장을 주었다	○	○	○	○	○	○	○	○

어제와 비교해서, 내 감기가 어떻다고 느껴집니까?

아주많이 좋아졌다	어느정도 좋아졌다	조금 좋아졌다	똑같다	조금 나빠졌다	어느정도 나빠졌다	아주많이 나빠졌다
○	○	○	○	○	○	○

- This is a 0-3 point scale for evaluating the severity of nasal congestion symptoms. A score of 0 means no symptoms, while a score of 3 indicates severe symptoms that are difficult to endure and interfere with daily life or sleep.
- To be eligible for this study, subjects must have a nasal congestion symptom score of 2 or higher at screening.
- Daily diary containing the nasal congestion symptom score will be distributed at Visit 2, and subjects will be instructed to self-evaluate daily. It will be collected at Visit 3.

Nasal Congestion Severity Score

Score	Criteria
0	No symptom
1	Symptom clearly present but minimal awareness
2	Definite awareness of symptom, which is bothersome but tolerable
3	Symptom that is hard to tolerate, may cause interference with activities of daily life or sleeping

10.3.2.4.Nasal Symptom Score VAS

- The severity of nasal symptoms (rhinorrhea, nasal congestion, sneezing) will be evaluated using a Visual Analogue Scale (VAS). The subject will mark their symptom severity on a scale where 0 represents no symptoms at all and 100 represents the most severe symptoms imaginable.
- Daily diary containing the Nasal Symptom Score VAS will be distributed at Visit 2, and subjects will be instructed to self-evaluate daily. It will be collected at Visit 3.

10.3.2.5.Systemic Symptom Score VAS

- The severity of systemic symptoms (headache, myalgia, chills) will be evaluated using a Visual Analogue Scale (VAS). The subject will mark their symptom severity on a scale where 0 represents no symptoms at all and 100 represents the most severe symptoms imaginable[15].
- Daily diary containing the Systemic Symptom Score VAS will be distributed at Visit 2, and subjects will be instructed to self-evaluate daily. It will be collected at Visit 3.

10.3.2.6.Duration of Cold Symptom

- Subjects will be asked if all cold-related symptoms have resolved. If they respond that the cold is completely gone, the date of resolution will be confirmed to assess the total duration of symptoms.
- Daily diary distributed at Visit 2 will be used to check for daily symptom resolution, and it will be collected at Visit 3.

10.3.2.7. **Recovery from Common Cold**

- At Visit 3, cold recovery will be evaluated based on whether all cold symptoms have improved.

10.3.2.8. **Global Evaluation of Efficacy**[16][17]

- This is a 0-4 point scale to assess the overall satisfaction with the treatment. The subject will self-evaluate (0=very good, 1=good, 2=average, 3=poor, 4=very poor).
- This will be assessed at Visit 3.

10.3.2.9. **EQ-5D-5L (5-level EuroQol 5-dimensional questionnaire)**

- The EQ-5D-5L is a self-reported questionnaire used to evaluate quality of life. It assesses five dimensions—mobility, self-care, usual activities, pain/discomfort, and anxiety/depression—on a 5-point scale. Additionally, subjects will mark their current health status on a vertical VAS ranging from 0 (worst imaginable health state) to 100 (best imaginable health state)[18].
- This will be assessed at Visits 2 and 3.

10.4. Safety Assessment

1) Adverse events
2) Vital signs
3) Laboratory test
   - ✓ Complete blood count: RBC, WBC, Hemoglobin, Hematocrit
   - ✓ Liver function test: AST, ALT, BUN, Creatinine, Glucose (Glucose will be evaluated only at Visit 1)

10.5. Exploratory outcome measures

10.5.1. Questionnaire for Common Cold Pattern Identification

- To examine the difference in effects by pattern identification, it will be used to classify subjects' patterns.
- It classifies pattern types into 8 categories: Wind-Cold type, Wind-Heat type, Dampness type, Summer-Heat type, Qi deficiency type, Blood deficiency type, Yin deficiency type, and Yang deficiency type[19]. In this study, the six pattern types other than Wind-Cold and Wind-Heat types will be classified as 'Other' to create a total of three pattern types (Wind-Cold type, Wind-Heat type, and Others).
- This will be assessed at Visit 1.

10.6. Adverse Events and Serious Adverse Events

10.6.1. Definition of Adverse Events

Adverse Event (AE) is any untoward and unintended sign (including abnormal laboratory findings), symptom, or disease that occurs in a subject administered an investigational product, and it does not necessarily have a causal relationship with the investigational product.

<Adverse Event Assessment>
Adverse events will be carefully monitored from the time the investigational product is administered. They will be confirmed during subsequent visits through interviews and medical consultations with the investigator. Detailed information will be recorded, including the onset and resolution dates of the adverse event, its severity, outcome, any actions taken in relation to the investigational product, causality with the investigational product, the name of any other suspect medications, and whether and how the adverse event was treated. In the event of an adverse event, subjects must be educated to report it immediately to the investigator and to visit the clinic for treatment. The investigator's contact information must be provided to the subjects. Detailed assessment, recording, and management of adverse events will follow the procedures in Section 10.6.3.

Adverse Drug Reaction (ADR) is any noxious and unintended response to an investigational product at any dose, where a causal relationship with the investigational product cannot be ruled out.

Unexpected ADR is an adverse drug reaction that differs in nature or severity from the information available in the Investigator's Brochure or other relevant drug information.

10.6.2. Definition of Serious Adverse Events

Serious Adverse Event / Adverse Drug Reaction (Serious AE/ADR) is an adverse event or adverse drug reaction occurring at any dose of an investigational product that leads to any of the following outcomes

- Death or life-threatening situation.
- Requires or prolongs hospitalization.
- Results in persistent or significant disability/incapacity.
- Causes a congenital anomaly or birth defect in an offspring.
- Other medically significant events, such as drug dependency or abuse, or hematological disorders, that are not listed above.

However, the following are not considered Serious AEs/ADRs

- Hospitalization for a simple observation or examination with no treatment.
- Subject's elective surgery or a pre-planned surgery for an existing condition.

Suspected Unexpected Serious Adverse Reaction (SUSAR) is any serious, unexpected, and suspected

adverse drug reaction that occurs during the use of an investigational product. It must meet all three of the following criteria

- Suspected adverse drug reaction
- Serious
- Unexpected

10.6.3. Classification of Adverse Events

1) Severity of Adverse Events

The severity of an adverse event is classified based on its maximum intensity using the following criteria

**Mild**	The subject barely notices it, and it does not interfere with normal daily life or functioning. It typically does not require treatment.
**Moderate**	The subject experiences discomfort and it interferes with normal daily life or functioning. The subject can continue the study, but treatment may be required.
**Severe**	The subject is very uncomfortable, daily life and functioning are impossible, and continued participation in the study is not feasible. Treatment or hospitalization may be required.

2) Causality Assessment of the Investigational Product

When an adverse event occurs, the investigator will assess its causal relationship with the investigational product based on the following criteria and provide their opinion if necessary.

A causal relationship with an investigational product cannot be statistically tested, so it is determined by considering individual circumstances, medical plausibility (physiological, pathological, pharmacological), and literature. Considering the temporal relationship is also helpful. Additionally, the investigator will consider whether the event disappeared upon discontinuation and reappeared upon re-administration of the drug, as well as any concomitant therapies or medications.

The certainty of the relationship between the adverse event and the investigational product (or other causes, such as underlying disease progression or concomitant treatment) is determined by how well the adverse event can be explained in terms of the following

① Known pharmacological actions of the investigational product.
② Similar actions previously observed with the investigational product or a drug of the same class.

③ Reactions frequently reported in relation to similar drugs.
④ A reaction that is temporally related to drug administration (disappears upon drug

discontinuation and reappears upon re-administration).

<Criteria for Causality Assessment with the Investigational Product>

**Definitely related**	- There is evidence of drug administration, and the temporal relationship between the investigational product and the adverse event is plausible.<br>- The adverse event is most plausibly explained by the investigational product rather than any other reason.<br>- The adverse event disappears when the investigational product is discontinued.<br>- The result of re-challenge (if performed) is positive.<br>- The adverse event shows a pattern consistent with previously known information about the same investigational product.
**Probably related**	- There is evidence of drug administration, and the temporal relationship between the investigational product and the adverse event is plausible.<br>- The adverse event is more plausibly explained by the investigational product than by other causes.<br>- The adverse event disappears when the investigational product is discontinued.
**Possibly related**	- There is evidence of drug administration, and the temporal relationship between the investigational product and the adverse event is plausible.<br>- The adverse event is judged to be attributable to the investigational product at the same level as other possible causes.<br>- The adverse event disappears when the investigational product is discontinued (if performed).
**Probably not related**	- There is evidence of drug administration, but there is a more plausible cause for the adverse event.<br>- The result of discontinuing the investigational product (if performed) is negative or ambiguous.<br>- The result of re-challenge (if performed) is negative or ambiguous.
**Definitely not related**	- The subject did not receive the investigational product.<br>- The temporal relationship between the investigational product and the adverse event is not plausible.<br>- There is another clear cause for the adverse event.
**Unknown**	- The information is insufficient or contradictory, and cannot be supplemented or confirmed.<br>- There is insufficient evidence to determine a connection.<br>- The quality of the evidence is poor or the data is inconsistent.

3) Predictable Adverse Events

- A predictable adverse event is any untoward and unintended reaction that occurs during an intervention, for which a causal relationship with the intervention used in the clinical trial cannot be excluded.

① GGTCS

1) Pseudoaldosteronism: May present with decreased urine output, swelling of the face and extremities, heavy eyelids, stiff hands, elevated blood pressure, and headache. Since formulations containing 1g or more of Glycyrrhizae Radix et Rhizoma per day may cause pseudoaldosteronism (hypokalemia, increased blood pressure, sodium retention, edema, weight gain) with long-term use, subjects must be closely monitored (by measuring serum potassium levels), and the medication should be discontinued if any abnormalities are found.<br>2) Myopathy: Myopathy may occur as a result of hypokalemia. Subjects should be closely observed, and the medication should be discontinued if abnormalities such as weakness, limb cramps, or paralysis are observed.<br>3) Skin: May cause rash, erythema, itching, etc.<br>4) Gastrointestinal System: May cause loss of appetite, stomach discomfort, nausea, vomiting, diarrhea, etc.<br>5) Autonomic Nervous System: May cause insomnia, excessive sweating, tachycardia, frequent urination, palpitations, systemic weakness, mental agitation, etc.<br>6) Urinary System: May cause micturition disorders.

10.6.4. Follow-up of Adverse Events

The Principal Investigator must continuously follow up on all collected adverse events until they are resolved or stable. Serious adverse events that have not resolved by the time a subject completes the study (at their last visit or date of discontinuation) must be followed up until one of the following occurs.

- The serious adverse event resolves or stabilizes (as per the investigator's clinical judgment).
- Abnormal clinical test values return to the normal range or baseline.
- The subject dies.
- Follow-up becomes impossible (e.g., loss of contact).

If a study must be prematurely terminated due to an adverse event, an adverse event report must be created and a final laboratory test conducted.

If a follow-up of a subject is impossible before an adverse event resolves/stabilizes or its cause is determined, the Principal Investigator must make every effort to contact the subject and collect information. This must also be documented.

10.6.5. Reporting of Adverse Events

During the study, the Principal and Sub-investigators must ensure the safety of the subjects. In the event of a serious adverse event, they must take swift and appropriate action.

In the event of a serious adverse event, the responsibilities of each person in charge are as follows:

1) Sub-Investigators

The sub-investigator must report all serious adverse events that occur during the study to the Principal Investigator within 24 hours, regardless of their relationship to the investigational product. Any other events considered serious by the sub-investigator or that suggest a significant risk, contraindication, side effect, or precaution related to the medication must also be recorded as a serious adverse event in the CRF.

2) Principal Investigator

The Principal Investigator must verbally or by email notify the assigned monitor at the Korea Institute of Oriental Medicine within 24 hours of becoming aware of any serious adverse event, in accordance with the Clinical Trial Management Guidelines. They must also report it to the Institutional Review Board (IRB) within the period set by the IRB.

Furthermore, a detailed written follow-up report must be submitted within 10 days of the serious adverse event's occurrence. In this case, to protect the confidentiality of the subject's personal information, the Principal Investigator must use a subject identification code instead of the subject's name, resident registration number, and address. The Principal Investigator must also suspend all or part of the clinical trial until further notice.

The following are not considered a serious adverse event:

- Hospitalization that is clearly unrelated to the investigational product.
- Hospitalization for simple observation or examination with no treatment.

In the event of a Suspected Unexpected Serious Adverse Reaction (SUSAR), the Principal Investigator must report it through the electronic reporting system for clinical trial adverse drug reactions on the MFDS e-civil application website (http://ezdrug.mfds.go.kr) within the deadlines stipulated by the Clinical Trial Management Guidelines.

- Events resulting in death or that are life-threatening: Within 7 days of the Principal Investigator receiving or becoming aware of the fact. However, if all information required by Form 77, such as the adverse drug reaction name, final observation results, and a summary of the adverse drug reaction, has not been reported, the Principal Investigator must submit an additional report with detailed information within 15 days of first receiving or becoming aware of the adverse drug reaction.
- Other serious and unexpected adverse drug reactions: Within 15 days of the Principal Investigator receiving or becoming aware of the fact.

When reporting an adverse drug reaction to the Minister of Food and Drug Safety, the principal

investigator must attach a summary of the adverse drug reaction, such as a CIOMS-I form, to the adverse drug reaction report. The investigator must also periodically report additional safety information until the adverse drug reaction is resolved (disappears or further follow-up is not possible).

For the assessment and reporting of a SUSAR, unblinding must be performed as necessary in accordance with the ICH E2A guideline, as long as it does not affect the conduct or outcome of the clinical trial. However, if an adverse drug reaction results in death, unblinding must be performed to confirm the type of suspected drug, and this information must be included in the SUSAR report.
A SUSAR report that has been unblinded must not be provided to or exposed to the blind team.

11. Data Analysis and Statistical Analysis

11.1. General Principles of Statistical Analysis

11.1.1. Definition of Analysis Sets

The efficacy analysis of this clinical trial will primarily use the Full Analysis Set (FAS), with the Per-Protocol Set (PPS) used for supplementary verification as needed.

1) FAS: Adhering to the Intention-To-Treat (ITT) principle, the FAS includes all randomized subjects who have taken the investigational product at least once and for whom efficacy data have been collected at least once. Data from subjects not meeting pre-established criteria will be excluded. The exclusion criteria for the FAS analysis are as follows:
    - Violation of inclusion or exclusion criteria
    - Subjects who did not take the investigational product even once
    - Subjects who did not provide any data after screening
2) PPS: The PPS consists of subjects from the FAS who have a medication compliance of 70% or more, have completed all procedures as specified in the protocol, and have no major protocol violations that could affect the results. The definition of major violations follows the details in Section 7.1 of this protocol.

The safety analysis will include data from all subjects who have taken the investigational product at least once after randomization and for whom a safety assessment was performed. The analysis will be conducted based on the group to which subjects were actually assigned.

11.1.2. General Principles of Analysis

1) All statistical analyses will be based on a two-sided test with a significance level (α) of 5%.
2) The statistical software SAS® Version 9.4 will be used for all analyses.

3) Continuous data will be presented as mean and 95% confidence interval, while categorical data will be presented as frequency and percentage.
4) If necessary, subgroup analysis will be conducted by categorizing subjects based on their baseline characteristics at the screening or baseline visit.

11.1.3. Handling of Missing Data

Missing data will be imputed using the Last Observation Carried Forward (LOCF) method. This method will only be applied to efficacy endpoints in the FAS.

11.1.4. Analysis of Demographic and Baseline Data

Descriptive statistics for demographic and pre-treatment subject characteristics will be presented by group. Continuous variables will be analyzed using an independent t-test or Wilcoxon rank sum test depending on normality. Categorical variables will be analyzed using a Chi-square test or Fisher's exact test.

11.2. Statistical Analysis of Outcome Measures

11.2.1. Primary Outcome Measures

Hypothesis: The mean change in the WURSS-21-K total score (symptom score + quality of life score) from baseline to Day 7 is not equal between the test group and the control group.

The analysis will be conducted using an ANCOVA model with the group as the effect and the baseline WURSS-21-K total score as a covariate. If necessary, other variables that show a statistical difference in demographic characteristics or that could affect cold symptoms will also be included as covariates. The least-square mean (LSM) difference between the control and test groups, its 95% confidence interval, and the p-value will be presented.

11.2.2. Secondary Outcome Measures

1) The change from baseline to Day 7 in Nasal Congestion Severity Score, Nasal Symptom Score VAS, Systemic Symptom Score VAS, WURSS-21-K symptom and quality of life scores, EQ-5D-5L score, and duration of cold symptoms will be tested using the same method as the primary outcome measures.
2) To compare the differences in trend changes between groups, a Repeated Measures Analysis of Variance (RM ANOVA) will be used, with Dunnett's procedure for multiple comparison correction (comparing each time point to baseline).
3) The Global Evaluation of Efficacy satisfaction and cold recovery status on Day 7 will be analyzed using a Chi-square test. If the Global Evaluation of Efficacy score is treated as a continuous variable, an Independent t-test or Wilcoxon rank sum test will be performed.

11.2.3. Exploratory Outcome Measures

The analysis of pattern identification will follow the analysis methods for the efficacy outcome measures.

11.3. Statistical Analysis of Safety Assessment

The safety evaluation will analyze the frequency of adverse events and serious adverse events suspected to be related to the treatment. Adverse events will be collected through subject reports or investigator observations. The reported adverse events will be described narratively, and the frequencies of events related and unrelated to the study intervention will be recorded and presented using descriptive statistics. The event count per visit between the groups will be compared using Fisher's exact test. The pre- and post-treatment values for each laboratory test variable will be analyzed using a paired t-test to determine if there is a difference.

11.4. Collection and Management of Clinical Trial Data

All information related to the clinical trial must be recorded, processed, and maintained to allow for accurate reporting, interpretation, and verification, in accordance with the Good Clinical Practice (KGCP) guidelines. The clinical trial site has the obligation to maintain and provide all essential clinical trial documents, including Case Report Forms (CRFs), signed consent forms, and source documents. Essential documents are all documents (including electronic documents) used for the individual or overall evaluation of the conduct of the clinical trial and the quality of the data obtained. The data entered in the CRF must match the source documents, and any discrepancies must be explained. When correcting or amending content in the CRF, the original content must remain legible, and the date and reason for the amendment must be documented and signed, following the correction guidelines. The principal investigator must comply with the Clinical Trial Management Guidelines and relevant laws and regulations regarding all aspects of data collection, recording, documentation, and reporting.

11.5. Storage and Confidentiality

11.5.1. Storage of Clinical Trial Documents and Data

A secure location will be prepared to store all documents and records related to the conduct of the clinical trial. To prevent premature damage or loss, documents and data will be stored in a locked area, and electronic data will be saved on a computer with restricted access to maintain security.
The Principal Investigator must retain essential documents and other clinical trial-related documents (including electronic documents) in accordance with relevant laws and regulations. However, after the final report is completed, these documents must be handed over to the custodian of records. The custodian of records at the study site must preserve these documents for a period of 3 years from the date of clinical trial completion.

11.6. Confidentiality of Clinical Trial Data and Subject Records

Subject records containing personal information must be handled in accordance with relevant regulations to ensure confidentiality. All records that could identify a subject must be kept confidential, and subjects will be managed and evaluated using a unique number and initials assigned at the start of the study. However, monitors, auditors, the Institutional Review Board (IRB), or the Ministry of Food and Drug Safety (MFDS) may directly review the subjects' medical records and clinical trial-related data to verify the procedures and data reliability of the trial, within the scope of relevant regulations and without infringing on subject confidentiality. Any individual or institution that directly reviews clinical trial-related data is obligated to maintain the confidentiality of the subject's identity and related information.

12. Ethical Considerations and Administrative Procedures

12.1. Subject Consent Procedures

Subject consent must be obtained in accordance with Article 30, Paragraph 1, Subparagraph 4, the ethical principles based on the Declaration of Helsinki, and these standards. Before the clinical trial begins, the principal investigator must obtain approval from the IRB for the consent form, the subject information sheet, and any other documented information provided to the subjects.

If new information related to the clinical trial that may affect a subject's consent is obtained, the consent form, subject information sheet, and other documented information must be revised accordingly. The revised forms and documents must be approved by the IRB before they are provided to the subjects. In this case, the principal investigator will inform the subject or the subject's representative in a timely manner and record the name of the person notified, the date of notification, and the content of the notification.

The principal investigator or a clinical trial staff member delegated by them must sufficiently inform the subjects about the clinical trial using the IRB-approved explanatory statement. They must provide sufficient time and opportunity for the subjects to ask questions about the clinical trial details and to decide on their participation. All clinical trial-related questions must be answered to the subject's satisfaction. Before the subject participates in the clinical trial, the subject and the principal investigator or a delegated clinical trial staff member who obtained the consent must sign the consent form and handwrite the date of consent.

12.2. Measures for the Ethical Conduct of the Trial and Protection of Subject Safety

This clinical trial will be conducted in compliance with all applicable regulations, including the Clinical Trial Management Standards and laws related to human subject research. Furthermore, this clinical trial will be conducted in accordance with the Declaration of Helsinki, respecting the dignity and rights of individuals and ensuring that no disadvantage is caused to the subjects.

12.2.1. Clinical Trial Site

The head of the clinical trial site must have the necessary facilities and professional personnel to conduct the clinical trial and must take all precautions to ensure the trial is conducted appropriately.

12.2.2. Approval and Amendment of the Clinical Trial Protocol

The clinical trial protocol must be reported to and approved by the Ministry of Food and Drug Safety and the IRB. No subjects may be enrolled in the clinical trial before the protocol is approved. An amendment to the clinical trial protocol must be made if the trial procedures are expanded, the risk increases, the subject selection criteria change, or additional safety information necessitates a change. When amending the clinical trial protocol, the revised plan must be approved by the Ministry of Food and Drug Safety and the IRB. The investigator must not conduct the clinical trial in a way that differs from the protocol, except when necessary to remove an immediate risk to a subject. The principal investigator or clinical trial staff must record all deviations from the approved clinical trial protocol and the reasons for them.

12.2.3. Familiarization with the Clinical Trial Protocol

The principal investigator and clinical trial staff must thoroughly understand and be familiar with the clinical trial protocol and conduct the trial in accordance with the protocol and relevant regulations.

12.3. Participant Compensation Policy

In the event of an injury resulting from participation in this clinical trial, appropriate and prompt compensation will be provided in accordance with the compensation standards and procedures set forth in the participant compensation policy and relevant laws. An 'injury resulting from participation in the clinical trial' means an injury caused by the investigational product administered according to the clinical trial protocol, or by a medical treatment process or intervention carried out according to the protocol, that would not have occurred had the subject not participated in the clinical trial.

12.4. Confidentiality of Subject Records

Records that can identify the subjects must be kept strictly confidential at the clinical trial site. Monitors and auditors related to this trial may access subject records for the purpose of monitoring, auditing, and managing the progress of this trial. This information must be kept confidential, and the facilities and management standards for maintaining confidentiality must be in place.

All documents related to the clinical trial, such as case report forms, must be recorded and identified by the subject identification code and initials, not the subject's name. For the purpose of statistical analysis

and scientific reporting, subject data will be stored in an electronic case report form, which will not include the subject's contact information or identifying details.

12.5. Protective Measures for Vulnerable Subjects

Vulnerable subjects are individuals whose voluntary decision to participate may be influenced by an expectation of benefits related to clinical trial participation or a fear of negative consequences from a superior in an organizational hierarchy if they refuse to participate. This includes students of medical, oriental medicine, pharmacy, dentistry, or nursing colleges; employees of medical institutions or research institutes; pharmaceutical company employees; military personnel; people in a direct hierarchical relationship with the researcher; people with incurable diseases; people in institutional care; unemployed persons; impoverished persons; patients in emergency situations; ethnic minorities; vagrants; homeless persons; refugees; minors; and individuals who cannot give consent of their own free will.

In this clinical trial, the participation of students from oriental medicine colleges, employees of medical institutions or research institutes, and unemployed persons is possible. The investigator will not engage in inducement activities to recruit these subjects and will allow their participation only if their participation is voluntary, within the scope of protecting their rights and welfare. To reconfirm their voluntary will, subjects will be asked to handwrite and sign their name and date on the consent form, explicitly stating, "I have voluntarily participated in this clinical trial".

When conducting a clinical trial on individuals affiliated with the research institution, the investigator must be especially strict about privacy and confidentiality. Measures must be taken to ensure that a refusal to participate is not disclosed to superiors, so the person is not unfairly influenced or coerced. Subjects should not be pressured or coerced, and they should be given sufficient time to understand the study.

12.6. Monitoring and Quality Control

Monitoring will be conducted to protect the rights and welfare of clinical trial subjects, to ensure that the reported clinical trial data are accurate, complete, and verifiable against source documents, and to confirm that the clinical trial is being performed in accordance with the approved protocol, Clinical Trial Management Standards, and their enforcement rules. Clinical trial monitoring may be conducted by a monitor designated by the Korea Institute of Oriental Medicine through regular visits to the clinical trial site or via telephone.

The scope of monitoring activities includes verifying compliance with the clinical trial protocol, ensuring proper and accurate data collection, checking subject consent and re-consent forms, collecting and reporting (serious) adverse events, and managing the investigational product. During a visit, the monitor will check the original source documents of clinical trial subjects, drug accountability records, and data storage. Clinical trial monitoring will be carried out according to a Monitoring Plan (MP), which is a

document describing the strategy, methods, responsibilities, and requirements for clinical trial monitoring. The monitor will check the progress of the clinical trial and consult with the investigator if any issues arise. The schedule for monitoring visits will be determined by mutual agreement between the investigator and the monitor. The investigator must allow the monitor to verify the clinical trial subject's source documents and essential documents that allow for the verification of the data entered in the case report forms, as defined in the Clinical Trial Management Standards and their enforcement rules.

12.7. Reporting of Trial Results

After the completion of the clinical trial, a clinical trial report detailing the purpose, design, methods, results, and statistical analysis of the trial must be prepared and submitted to the IRB and the Ministry of Food and Drug Safety.

12.8. Publication of Trial Results

The trial results, in whole or in part, may not be published or used without the consent of the Korea Institute of Oriental Medicine.

13. Other Necessary Matters for the Safe and Scientific Conduct of the Clinical Trial

13.1. Compliance with Laws, Audits, and Prohibitions

This clinical trial will be conducted in accordance with the clinical trial protocol (including the subject information sheet and consent form) approved by the Ministry of Food and Drug Safety and the IRB of the clinical trial site. The investigator must apply for approval or report changes to the clinical trial protocol to the Ministry of Food and Drug Safety and the IRB of the clinical trial site in advance, except in cases where the change is necessary to immediately prevent harm to the subject. If a change is implemented before obtaining approval from the Ministry of Food and Drug Safety and the IRB to immediately prevent harm to a subject, these changes must be reported to the Ministry of Food and Drug Safety and the IRB as soon as possible.

By signing the protocol, the investigator agrees to perform the trial effectively and faithfully in accordance with this clinical trial protocol, the regulations of the Good Clinical Practice (GCP), and all relevant domestic laws, including the "Rules on the Safety of Pharmaceuticals, etc. [Prime Minister's Decree No. 1353]" and regulations related to the conduct of clinical trials. The clinical trial staff and participating researchers must accurately analyze and familiarize themselves with the research plan. The principal investigator must take advance measures, such as providing sufficient training to the participating researchers, preparing for unexpected adverse events, and reporting them as required. The conduct of the clinical trial shall be in line with the Good Clinical Practice (GCP).

The investigator must accurately prepare, manage, and complete clinical trial-related documents in accordance with Good Clinical Practice (GCP) and all relevant domestic laws, regulations, and rules.

13.2. Research Data Sharing Plan

The personal information collected in this study (hereinafter referred to as "research information") may be provided to a third party in accordance with Article 18 of the Bioethics and Safety Act. For this purpose, the researcher will explain the provision of research information to third parties and obtain consent from the subjects through the <Notice and Consent Form for Third-Party Provision and Secondary Use of Research Information>. If the subject consents, the research information may be anonymized and provided to data processing institutions, academic societies, and human subject researchers, etc., after review by the institutional committee. The provided research information may be used for secondary research related to health care for purposes other than the original research purpose and will be used for statistical purposes according to each research objective. The items of research information to be provided are specified in the consent form, and a refusal to consent to secondary use of the research does not limit participation in the study.
Furthermore, for the research information of subjects to be used for secondary research purposes other than the original research purpose, the subject must be informed of the following matters and give their consent in accordance with Articles 17 and 18 of the Personal Information Protection Act. If any of the following items change, the subject must be informed and their consent obtained again.

14. References

[1] Benson V, Marano MA. Current estimates from the National Health Interview Survey, 1995. Vital Health Stat 10. 1998;(199):1-428.

[2] Health Insurance Review and Assessment Service. 2023 Frequent Disease Statistics. https://opendata.hira.or.kr/op/opc/olapHifrqSickInfoTab1.do. 2024.08.22

[3] Henneicke-von Zepelin, H. H., et al. Efficacy and safety of a fixed combination phytomedicine in the treatment of the common cold (acute viral respiratory tract infection): results of a randomised, double blind, placebo controlled, multicentre study. Current medical research and opinion, 1999. 15(3): p. 214-227.

[4] Heikkinen, Terho, and Asko Järvinen. The common cold. The Lancet, 2023. 361(9351): p. 51-9.

[5] Bok-Hyun Jung. Upper Airway Infection and Acute Bronchitis In: The Korean Academy of Tuberculosis and Respiratory Diseases editor. Pneumology. Seoul: Koonja Publishing Co.; 2004. p. 698

[6] Clinical Practice Guideline of Korean Medicine for Common Cold. Clinical Practice Guideline of Korean Medicine Development Group. 2021

[7] Cho, D., et al. Effect of Gal-Geun-Tang on antigen-specific immune response. The Journal of Korean Medicine Ophthalmology and Otolaryngology and Dermatology, 2016. 29(3): p. 134-49.

[8] Yang, TK, Kim YB, and Chae BY. An Experimental study on the anti-allergic effects, anti-inflammatory

action, anti-pyretic action and analgesic action of Galgeun-tang, Gamigalgeun-tang and Geomahwanggalgeun-tang. The Journal of Korean Medicine Ophthalmology and Otolaryngology and Dermatology, 2022. 15(1): 76-95.

[9] Kim, J. H., et al. Analysis of the use of insured herbal extracts and korean medicinal treatments in patients with allergic rhinitis: Data from health insurance review and assessment service. The Journal of Korean Medicine Ophthalmology and Otolaryngology and Dermatology, 2021. 34(2): p. 38-52.

[10] Committee for Textbook of Herbal Pharmacology for Korean Medicine Colleges. Herbal Pharmacology. Seoul: Younglimsa; 2003. 166-8, 447-8.

[11] Yamagiwa, M., The Immediate Effect of Kakkon-to-ka-senkyu-shin'i (Ge-gen-tang-jia-chuan-xiong-xin-yi) on Nasal Obstruction Evaluated by Acoustic Rhinometry. Kampo Medicine 46.1 (1995): 83-89.

[12] Eccles, R., Eriksson, M., Garreffa, S., & Chen, S. C. (2008). The nasal decongestant effect of xylometazoline in the common cold. American journal of rhinology, 22(5), 491-496.

[13] Barrett, Bruce, et al. Validation of a short form Wisconsin upper respiratory symptom survey (WURSS-21). Health and quality of life outcomes 7 (2009): 1-20.

[14] Yang, Su-Young, et al. Reliability and validity of Wisconsin upper respiratory symptom survey, Korean version. Journal of epidemiology 21.5 (2011): 313-318.

[15] Eccles, Ronald, et al. "Efficacy and safety of iota-carrageenan nasal spray versus placebo in early treatment of the common cold in adults: the ICICC trial." Respiratory research 16 (2015): 1-11.

[16] Analgesic and Decongestant Efficacy ofthe Combination of Aspirin With Pseudoephedrine in Patients With Symptoms of Upper RespiratoryTract Infection

[17] Michalsen, Andreas, et al. "The impact of cineole treatment timing on common cold duration and symptoms: Non-randomized exploratory clinical trial." Plos one 19.1 (2024): e0296482.

[18] Kim SH, Ahn JH, Ock MS, Shin SJ, Park JY, Luo N, et al. The EQ-5D-5L valuation study in Korea. Quality of life research. 2016;25(7):1845-1852.

[19] Byun, Jun-Seop, et al. Primary study to develop the instrument of pattern identification for common cold. Journal of Physiology & Pathology in Korean Medicine 23.6 (2009): 1226-1233.
